# The effects of applying cellulase and laccase on fermentation quality and microbial community in mixed silage containing corn stover and wet brewer’s grains

**DOI:** 10.3389/fpls.2024.1441873

**Published:** 2024-09-25

**Authors:** Li Li, Xiangxue Xie, Guoqiang Zhao, Jiajun He, Yongliang Zhang

**Affiliations:** ^1^ Guangdong Provincial Key Laboratory of Animal Nutrition Control, College of Animal Science, South China Agricultural University, Guangzhou, Guangdong, China; ^2^ Ruminant Product Research and Development Department, Guangdong VTR Bio-Tech Co., Ltd., Zhuhai, China

**Keywords:** cellulase, laccase, fermentation quality, microbial community, corn stover, wet brewer’s grains

## Abstract

**Objective:**

The purpose of this experiment was to explore the effect of adding cellulase and laccase on fermentation quality and microbial community in mixed silage of corn stover and wet brewer’s grains. Try to a new approach for the proper preservation and utilization of the agro-industrial by-products (corn stover and wet brewer’s grains).

**Methods:**

The experiment was divided into four groups: CK (control), C (cellulase, 120 U/g fresh matter [FM]), L (laccase, 50 U/g FM), CL (cellulase 120 U/g FW and laccase 50 U/g FM), and the chemical composition, fermentation quality, microbial population and microbial community in mixed silage of corn stover and wet brewer’s grains after 30 day’s fermentation were determined.

**Results:**

Compared to control, the addition of cellulase significantly increased crude protein (CP), water-soluble carbohydrate (WSC), lactic acid bacteria (LAB) counts, while significantly decreased the neutral detergent fiber (NDF), acid detergent fiber (ADF) content (*P* < 0.05). Adding laccase significantly decreased the acid detergent lignin (ADL) content (*P* < 0.05). Combined application of cellulase and laccase significantly increased the CP, WSC content and LAB counts, while significantly decreased pH value, NDF, ADF and ADL content (*P* < 0.05), thereby improving fermentation quality. In addition, the application of cellulase and laccase increased the abundance of Firmicutes and LAB genera, and decreased microbial diversity level of the sample.

**Conclusion:**

The combined application of cellulase and laccase further improved fermentation quality and microbial community in mixed silage of corn stover and wet brewer’s grains.

## 1 Introduction

Corn stover is one of the most common agricultural by-products in the world. China is the world’s second largest corn grower, with an annual corn stover output exceeding 200 million tons, accounting for a total crop straw output of 39% (Shi et al., 2016). In most cases, corn stover is incinerated; this is a significant waste of resources and generates a source of environmental pollution (Sun et al., 2019). Thus, the production of silage from corn stover not only reduces environmental pollution, but also alleviates problems caused by a lack of agricultural feed (He et al., 2019). However, the quality of corn stover silage is limited by its low digestibility and nutritional value; furthermore, this type of silage cannot fulfil the daily nutritional requirements of animals when provided as the raw material in a single food source. This limitation arises from a high content of lignocellulose and hemicellulose, and a low content of protein, amino acids, and minerals. These conditions are not conducive to the efficient growth of microorganisms. As an alternative, mixed silage is an effective method that can be used to improve the fermentation quality, nutritional value, and palatability, of corn stover silage (Ni et al., 2018).

Wet brewer’s grains are a by-product of the energy industry and brewing industry, have a high nutritional value, and are rich in crude protein (CP) and vitamin B (Anderson et al., 2019). These grains can be used as an ingredient for animal feeds as a good source of nutrients and minerals, especially for ruminant species (Stemme et al., 2005; Lo´ pez-Campos et al., 2011). However, the high moisture content of wet brewer’s grains renders this material unsuitable for storage. Another factor that limits the use of this by-product is its high fiber content. The long-term and sustainable utilization of wet brewer’s grains can be achieved by mixing wet brewer’s grains with materials that have a high dry matter (DM) content; this practice can improve the quality of silage fermentation (Chiou et al., 2000).

Ensiling is a practical method used to preserve high moisture forage that is based on lactic acid bacteria (LAB) anaerobically converting water-soluble carbohydrates (WSCs) into organic acids, thus reducing the pH and inhibiting the activity of organisms that can cause spoilage (Filya et al., 2000; Mcdonald et al., 1991). In addition, this practice can improve palatability as anti-nutritional factors are likely to be eliminated during the ensiling process.

Various biological additives have been used to enhance fermentation in silages; these additives are convenient, safe, non-corrosive, environmentally friendly, and regarded as natural products (Basmacioglu et al., 2003; Tian et al., 2020). Exogenous cellulase has been proven to degrade structural carbohydrates to soluble sugars, a substrate that is required for LAB fermentation (Colombatto et al., 2004; Wang et al., 2002). According to a recent report, mixing corn straw and soybean residue with 100 U/g of cellulase had better silage effect (Zhao C. et al., 2021). Laccase can catalyze the oxidation of phenol groups to degrade lignin (Qiu and Chen, 2012). Previous research has confirmed that laccase is an important enzyme involved in lignin degradation, a process that can improve the efficiency of cellulose degradation (Yang et al., 2019; Arias et al., 2016). During the silage process, microorganisms compete with each other for nutrients. Lactic acid bacteria predominantly use substrates, such as WSC, to produce lactic acid and become the most dominant group of bacteria. The growth and proliferation of lactic acid bacteria is a very important factor that determines the fermentation quality of silage. Next-generation sequencing (NGS) is generating new information regarding the microbial dynamics of ensiling forage (Guan et al., 2018), providing opportunities for further modulating silage fermentation.

It is hypothesized that both cellulase and laccase exert a positive effect on the fermentation characteristics and microbial communities of mixed silage created from corn stover and wet brewer’s grains. However, recent studies of stover silage have mainly focused on fermentation characteristics arising from the use of cellulase and LAB inoculants (Liu et al., 2016; Mu et al., 2020). Notably, little is currently known about the effects of enzyme preparation on the fermentation quality of mixed silage prepared from corn stover and wet brewer’s grains. The objective of this study was to investigate the nutritional and fermentation quality, and microbial communities, of silage prepared from agro-industrial by-products (corn stover and wet brewer’s grains) after ensiling with cellulase and laccase for 30 days (d).

## 2 Materials and methods

### 2.1 Silage preparation

Corn was planted on the 15^th^ of and harvested on the 5^th^ October 2021 in an experimental trials field maintained by VTR Bio-Tech Co., Ltd., (Doumen District, Zhuhai, Guangdong, China), located at 22°8′19″ N, 113°14′6″ E. Corn stover was the post-harvest residue of corn, which cut into 2 to 3 cm lengths. Wet brewer’s grains were purchased from a private beer company (Zhujiang Beer Co., Ltd., Guangzhou, China). The chemical composition of the raw material before ensiling is shown in **Table 1**. Corn stover and wet brewer’s grains were combined in a 2:3 ratio, thoroughly mixed and treated as follows: (1) control (CK); (2) cellulase (C; 120 U/g fresh matter [FM], Guangdong VTR Bio-Tech Co., Ltd., Zhuhai, China); (3) laccase(L; 50 U/g FM, Guangdong VTR Bio-Tech Co., Ltd., Zhuhai, China); (4) cellulase + laccase (CL; C 120 U/g FM + L 50 U/g FM). According to the specific experimental design, the enzyme preparations used for each treatment were dissolved in an equivalent volume of distilled water, mixed and homogenized with the raw material, and then transferred into polyethylene bags (24 × 40 cm), which were then vacuum sealed. An equal volume of distilled water was applied for control group. In this study, we used 12 bags in total (4 treatments × 3 replicates), which were maintained at an ambient temperature (-30°C). The bags were opened after ensiling for 30 days, silage was collected and sub-samples were collected. Samples for chemical composition analysis were dried and tested as described in the next section; samples for fermentation characteristics, microbial population, and community analysis, were stored at -80°C until required.

**Table 1:** Characteristics of silage materials.

Raw material	DM	CP	NDF	ADF	ADL	WSC
	g/kg FM	g/kg DM				
Corn straw	891.65	86.01	577.65	303.97	52.01	66.37
Wet brewer’s grains	190.64	242.20	540.87	343.42	153.97	25.70
Mixed materials	468.32	123.43	573.07	316.05	86.55	53.13

Mixed silage: corn stover and wet brewer’s grains (w:w=2:3); DM, dry matter; CP, crude protein; NDF, neutral detergent fiber; ADF, acid detergent fiber; ADL, acid detergent lignin; WSC, water-soluble carbohydrate; FM, fresh matter.

### 2.2 Chemical composition analysis

On day 30, one sub-sample of silage was oven-dried for 72 h at 65°C and the DM contents were calculated. Dried samples were ground and passed through a 1 mm mesh sieve and then stored in sealed vinyl bags to await chemical composition analysis. CP was determined using methods described by the Association of Official Analytical Chemists (AOAC, 2005) using a K9860 Kjeldahl Analyzer (Hanon Advanced Technology Group Co., Ltd., Jinan, China). The Van Soest method was used to assess neutral, acid detergent fiber and acid detergent lignin (NDF, ADF and ADL respectively) (Van Soest et al., 1991), for this method, we used an ANKOM 2000 Automated Fiber Analyzer (Ankom Technologies, Inc., Fairport, NY, USA). The sulfuric acid-anthrone colorimetric method was used to determine the content of WSC (Owens et al., 1999).

### 2.3 Fermentation characteristics

First, 20 g of each sample was placed in 180 mL of distilled water, mixed thoroughly, and left overnight at 4°C. Next, the mixture was filtered through four layers of gauze, and then filtered with qualitative filter paper. The filtrate was used to determine the fermentation characteristics. Silage acidity was determined using an AB 150 pH meter (Fisher Scientific International, Inc., Pittsburgh, PA, USA). The phenol-sodium hypochlorite colorimetric method (Weatherburn, 1967) was used to determine the content of ammonia nitrogen (NH_3_-N). Lactic acid (LA) was determined by high performance liquid chromatography (HPLC); acetic acid (AA), propionic acid (PA) and butyric acid (BA) were measured by gas chromatography (GC) (Zhao G. Q. et al., 2021).

### 2.4 Microbial population analysis

According to the spread-plate method (Madigan et al., 2012), 20 g of each sample was immediately blended with 180 mL of sterilized saline water (8.5 g/L NaCl), and serially diluted from 10^−1^ to 10^−8^. The number of LAB, *Escherichia coli* (*E.coli*), yeast and mold were incubated and counted using De Man, Rogosa and Sharpe agar, Violet Red Bile agar and Rose Bengal agar, respectively.

### 2.5 Microbial community analysis

Total DNA was extracted from the microbiome of silage samples with a specialized kit (D4015, Omega Inc., Norcross, GA, United States), and the high variation region of the bacterial 16S rRNA gene V3 ~ V4 was obtained with the pre-primer and post-primer. The amplified products were recovered and purified, and a library was sequenced by the Illumina NovaSeq sequencing platform. After sequencing, FLASH (version 1.2.11) was used to assemble raw reads, the QIIME (Version 1.7.0) quality control process was used to exclude low-quality reads and the UCHIME algorithm was used to remove chimeric sequences and obtain final effective tags (Mu et al., 2020). Effective tags were then clustered into operational taxonomic units (OTUs) at a 97% similarity level. According to the OTU results, alpha (Shannon, Simpson, Chao1, Ace and coverage) and beta diversity (Principal component analysis [PCA]) were derived from QIIME and R software (Version 2.15.3; R Foundation for Statistical Computing, Vienna, Austria), respectively. Microbial relative abundance was used to represent bacterial classification. We used linear discriminant analysis effect size (LEfSe) to identify significant associations between bacterial taxa in the treatments (Segata et al., 2011).

### 2.6 Statistical analysis

The effects of C, L and their combination on fermentation quality and microbial community in mixed silage of corn stover and wet brewer’s grains were analyzed using the IBM SPSS Statistics for Windows, version 23.0 (IBM Corp., Armonk, N. Y., USA). Duncan’s comparisons were used to identify differences between means, and identify differences between means, and *P* < 0.05 was considered significant. In addition, we used the Novomagic online platform (available at http://magic.novogene.com; Novogene Co., Ltd., China) to analyze microbial diversity.

## 3 Results

### 3.1 Chemical composition after ensiling

The chemical composition of silages after 30 d of ensiling is shown in **Table 2**. No two-way interaction of C×L was noted for any of parameters. Generally, the addition of cellulase exerted a significant effect on CP, NDF, ADF and WSC (*P* < 0.05), while laccase had a significant effect on CP and ADL content (*P* < 0.05). There was no significant difference in DM content among all groups (*P* > 0.05), The CP content in the CL group was significantly higher than that in all other groups, and the content of CP in the C group was also significantly higher than in the CK and L groups (*P* < 0.05). The WSC content of treated groups was higher than that of control group (by 144.17%, 63.85% and 129.69%, respectively). Furthermore, the WSC of C and CL was significantly higher than that of control group (*P* < 0.05). The NDF, ADF and ADL contents of all experimental groups were lower than that of control group, NDF and ADF were significantly different in the C and CL groups (*P* < 0.05). The ADL of group L and CL was significantly lower than in group C and CK (*P* < 0.05).

**Table 2:** Chemical composition of mixed silage of corn stover and wet brewer’s grains after ensiling.

Item		DM	CP	NDF	ADF	ADL	WSC
		g/kg	g/kg DM				
CK		455.92	114.75^c^	509.27^a^	289.01^a^	76.26^a^	9.60^b^
C		461.57	120.04^b^	458.67^b^	256.68^b^	72.06^a^	23.44^a^
L		452.90	115.37^c^	503.50^a^	279.60^a^	64.78^b^	15.73^ab^
CL		454.59	123.79^a^	453.51^b^	255.78^b^	62.16^b^	22.05^a^
SEM		3.368	0.390	7.401	1.667	1.009	1.029
*P*-value	C	0.601	<0.001	0.003	0.001	0.129	0.001
	L	0.479	0.028	0.716	0.138	0.001	0.281
	C*L	0.776	0.080	0.984	0.217	0.705	0.104

CK, no additives; C, added cellulase; L, added laccase; CL, cellulase and laccase; DM, dry matter; CP, crude protein; ADF, acid detergent fiber; NDF, neutral detergent fiber; ADL, acid detergent lignin; WSC, water-soluble carbohydrate.

### 3.2 Fermentation quality after ensiling

As shown in **Table 3**, the addition of cellulose and laccase significantly affects pH (*P* < 0.05). The pH of CL group was significantly lower than that of other groups (*P* < 0.05). The contents of lactic acid and acetic acid in the experimental groups were higher than that in control group, although there was no significant difference between the groups (*P* > 0.05). Propionic acid and butyric acid were not detected in any of the samples. The lactic acid/acetic acid values of C, L and CL were 25.53%, 2.84% and 2.84% higher than those of the control group, respectively, although there was no significant difference between the groups (*P* > 0.05). The levels of ammonia nitrogen/total nitrogen (NH_3_-N/TN) in the experimental groups were lower than those in the control group, although there was no significant difference between the groups (*P* > 0.05).

**Table 3:** Effects of applying cellulase and laccase on fermentation characteristics of corn stover and wet brewer’s grains after ensiling.

Group		pH	LA	AA	PA	BA	NH_3_-N g/kg TN	LA/AA
			g/kg DM					
CK		4.19^a^	15.92	10.70	ND	ND	62.59	1.41
C		4.16^a^	22.07	14.08	ND	ND	56.91	1.77
L		4.18^a^	16.56	10.60	ND	ND	60.26	1.45
CL		4.11^b^	16.85	18.74	ND	ND	50.43	1.45
SEM		0.005	1.021	1.333	–	–	1.785	0.179
*P*-value	C	0.001	0.166	0.074	–	–	0.066	0.692
	L	0.019	0.306	0.425	–	–	0.257	0.770
	C*L	0.128	0.201	0.407	–	–	0.580	0.692

CK, no additives; C, added cellulase; L, added laccase; CL, cellulase and laccase; NH_3_-N, ammonium nitrogen; TN, total nitrogen; LA, lactic acid; AA, acetic acid; PA, propionic acid; BA, butyric acid.

The effects of additive application on the microbial population of corn stover and wet brewer’s grains after ensiling are shown in **Table 4**. The addition of cellulase exerted a significant effect on LAB (*P* < 0.05). The LAB of CL was significantly higher than the CK and L groups (*P* < 0.05) and C group was significantly higher than that in the control group (*P* < 0.05). Yeast, mold and *E.coli* were not detected in any of the samples.

**Table 4:** Effects of applying cellulase and laccase on microbial population of corn stover and wet brewer’s grains after ensiling.

Group		LAB	*E.coli*	Mold	Yeast
		log CFU/g of FM			
CK		8.39^c^	ND	ND	ND
C		8.68^ab^	ND	ND	ND
L		8.47^bc^	ND	ND	ND
CL		8.70^a^	ND	ND	ND
SEM		0.035	–	–	–
*P*-value	C	0.006	–	–	–
	L	0.799	–	–	–
	C*L	0.980	–	–	–

CK, no additives; C, added cellulase; L, added laccase; CL, cellulase and laccase; LAB, lactic acid bacteria; E.coli, Escherichia coli; CFU, colony forming units; ND, not detected.

### 3.3 Venn analysis and alpha diversity indices of microbial community

**Figure 1:**
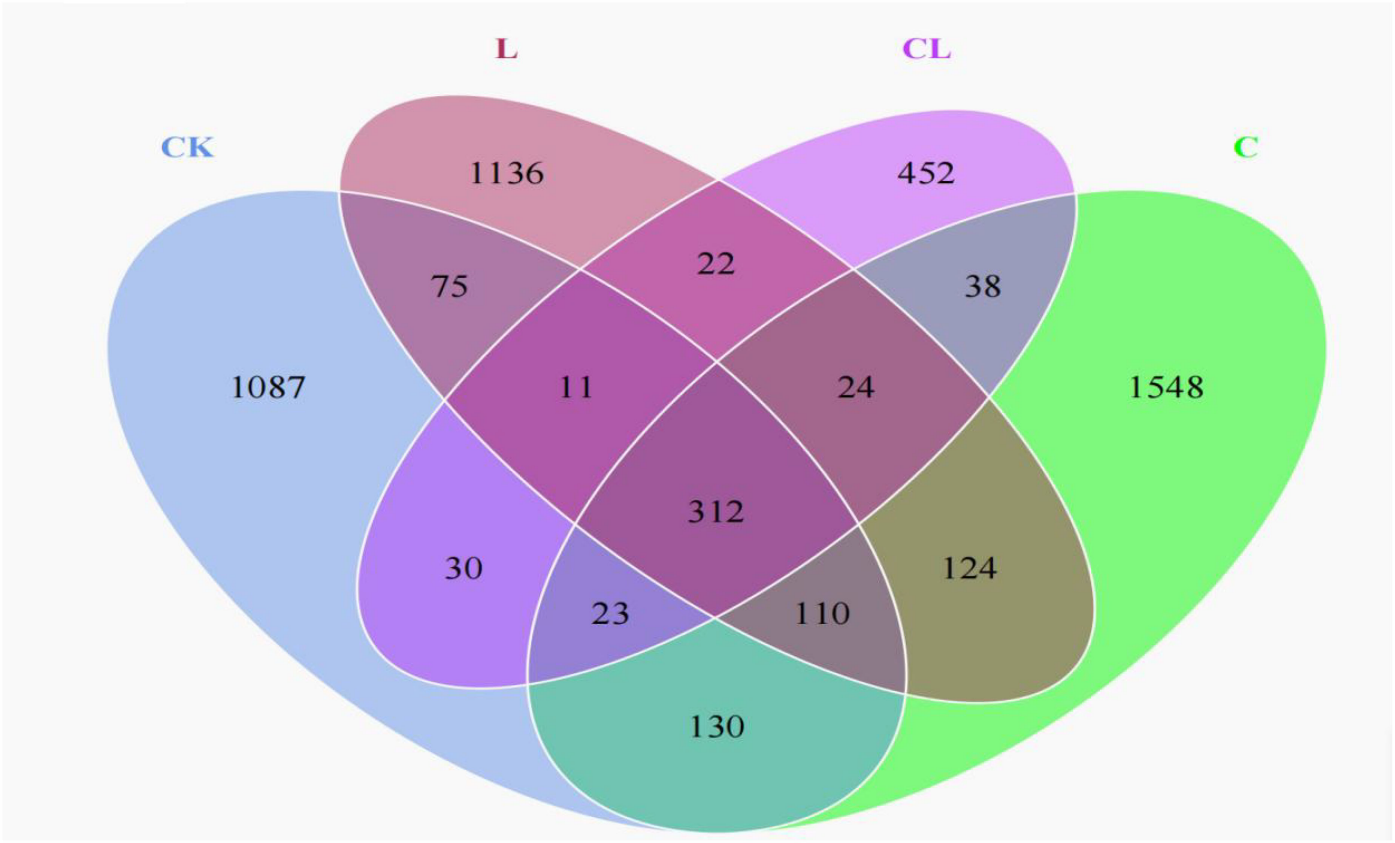
Venn diagram of bacterial OTUs of corn stover and wet brewer’s grains after ensiling.

The Venn analysis showed the shared and unique operational taxonomic units (OTUs) among all groups (**Figure 1**). Analysis identified 1087, 1548, 1136, and 452 unique OTUs in the CK, L, C and CL silages, respectively, with 312 OTUs being shared across all groups.

As shown in **Table 5**, goods coverages of all silage samples were higher than 99%, thus indicating good feasibility with regards to analyzing the microbial community. A lower number of OTUs, and lower Shannon, Simpson, Chao1 and Ace indices, were observed in CL silage when compared to CK, L and C silage after 30 d of ensiling.

**Table 5:** Alpha Diversity index of experimental group.

Group	Observed species	Goods coverage	Shannon	Simpson	Chao1	Ace
CK	804	0.996	3.476	0.740	919.229	980.452
C	992	0.996	3.512	0.739	1068.965	1124.759
L	781	0.996	3.124	0.723	890.805	944.091
CL	408	0.997	2.388	0.715	486.331	531.429

### 3.4 Principal component analysis of the microbial communities in each experimental group

**Figure 2:**
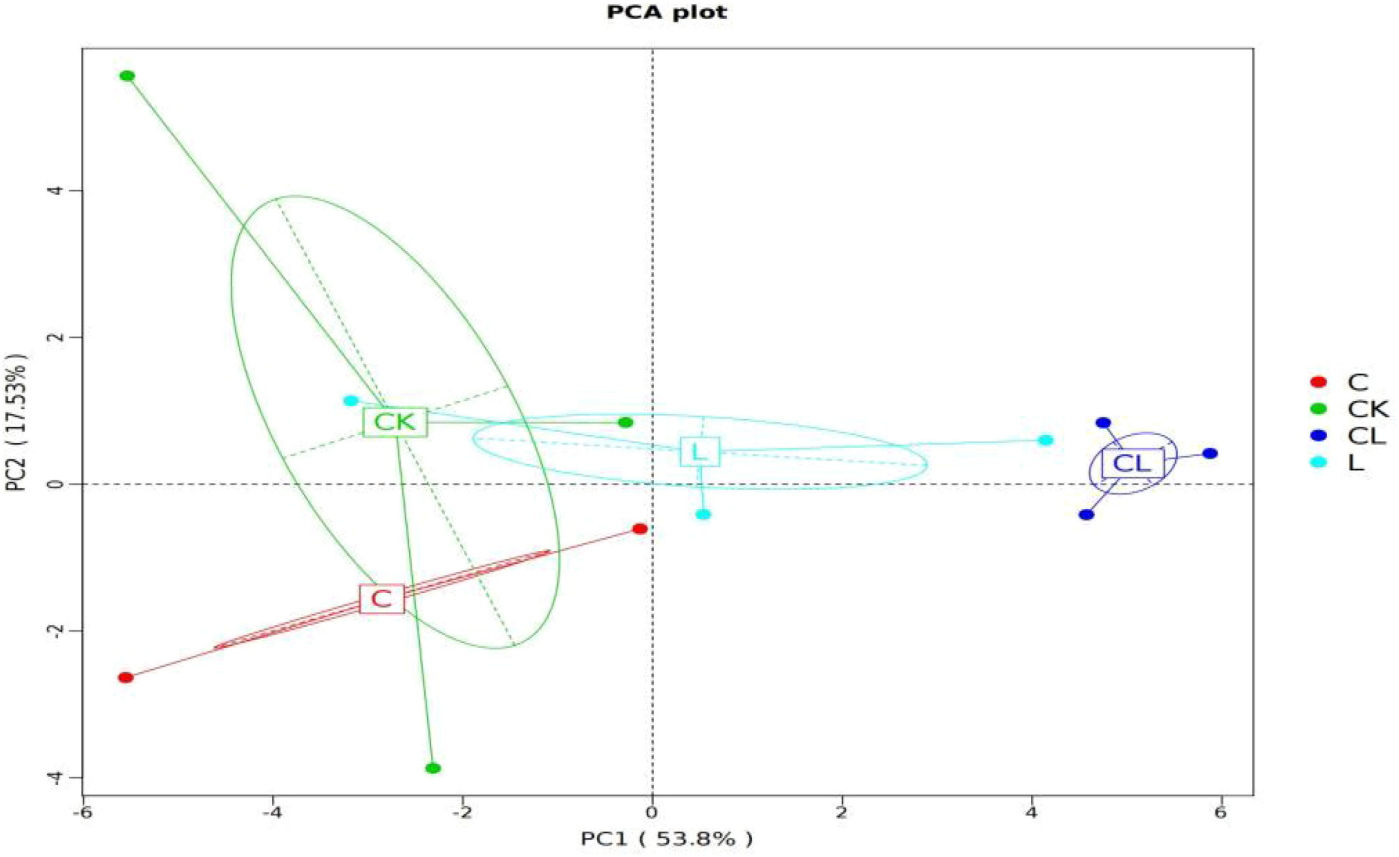
3D Sorting Chart of PCA Analysis.

The results arising from PCA based on OTU levels are shown in **Figure 2**. The CK group was clustered in the third and fourth quadrants, CL group was clustered in the first and second quadrants. Therefore, we inferred that there were distinctions among CK and CL groups in their bacterial communities. The contribution of component 1 and component 2 to the difference in results was 53.58% and 17.35%, respectively.

### 3.5 The effect of relative abundance on microbial community

**Figure 3:**
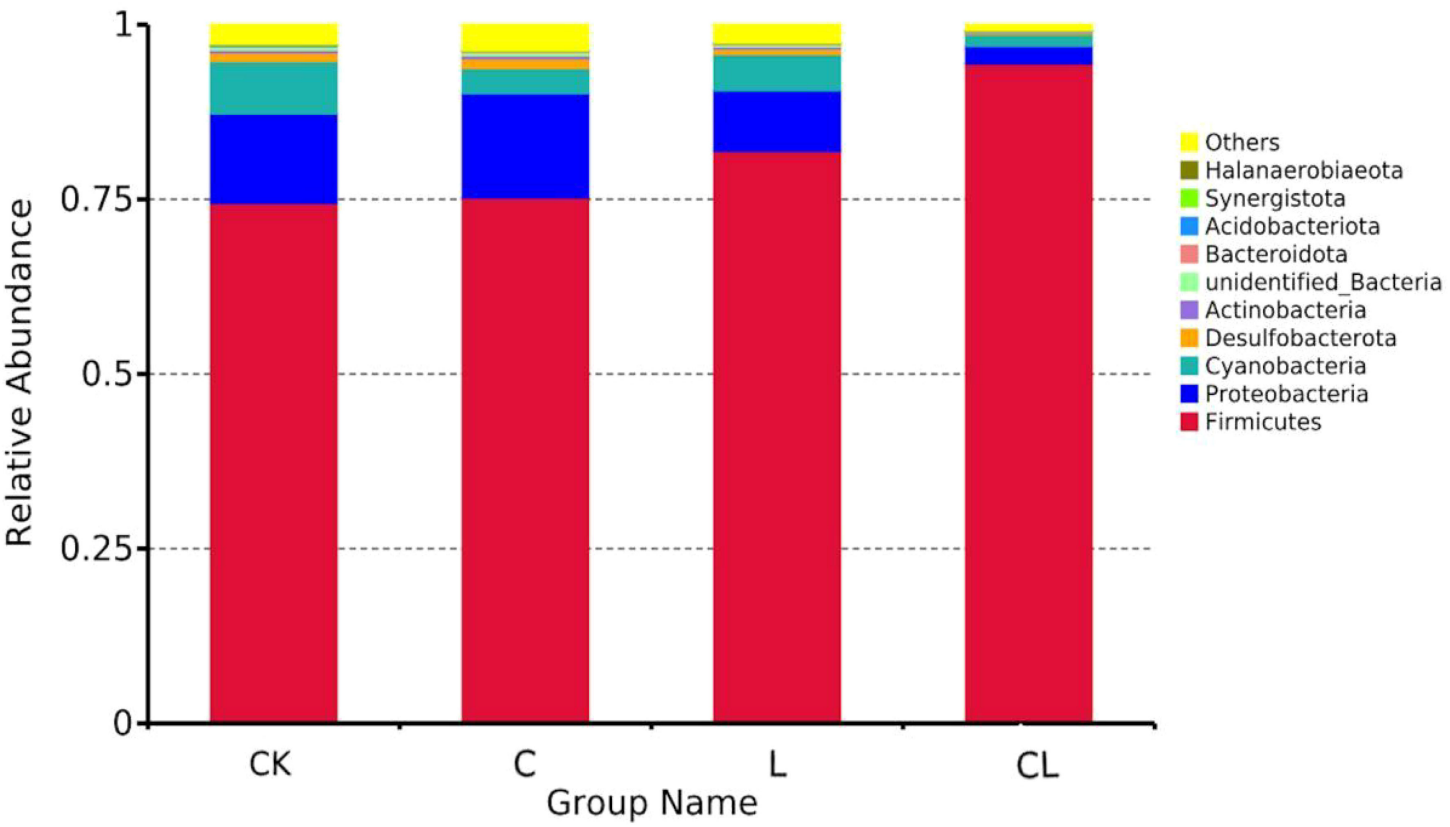
The histograms of abundances of microbes at phylum level in each silage group.

Bacterial communities (at the phylum level) in fresh material and in 30 d silage are shown in **Figure 3**. Firmicutes and Proteobacteria were the dominant phyla in mixed silages. Of these, Firmicutes was the most abundant, accounting for 74.48% (CK), 75.25% (C), 81.92% (L), and 94.43% (CL), respectively, in the four groups. Proteobacteria accounted for 12.75% (CK), 14.93% (C), 8.68% (L), and 2.51% (CL) in this study.

**Figure 4:**
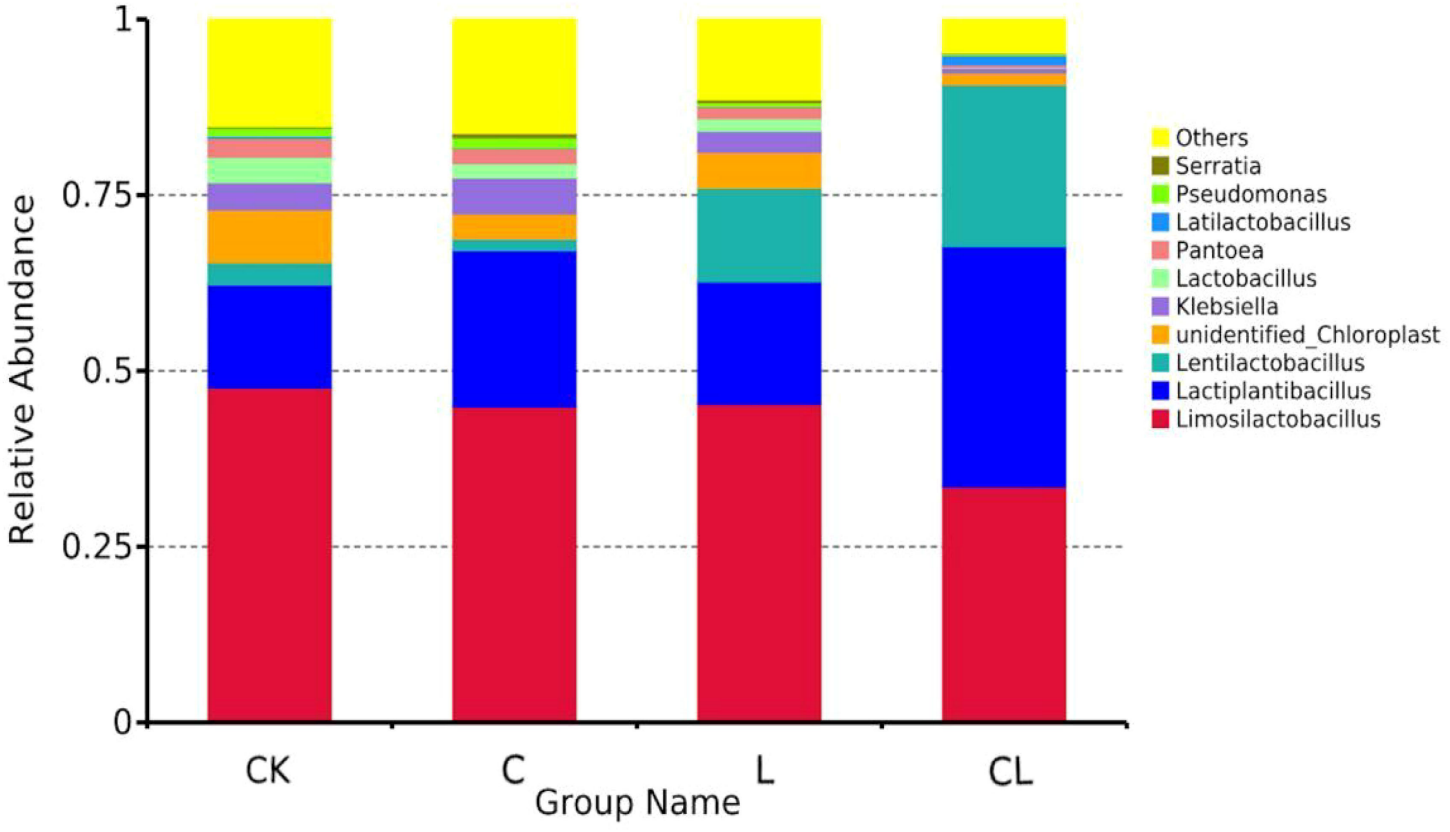
The histograms of abundances of microbes at genus level in each silage group.

The bacterial communities (at the genus level) in 30 d silages are in shown **Figure 4**. For all silages, *Limosilactobacillus, Lactiplantibacillus*, and *Lentilactobacillus* were the top three genera. The total abundance of these three genera in the four groups was 65.45%, 68.84%, 76.04%, and 90.75%, respectively.

**Figure 5:**
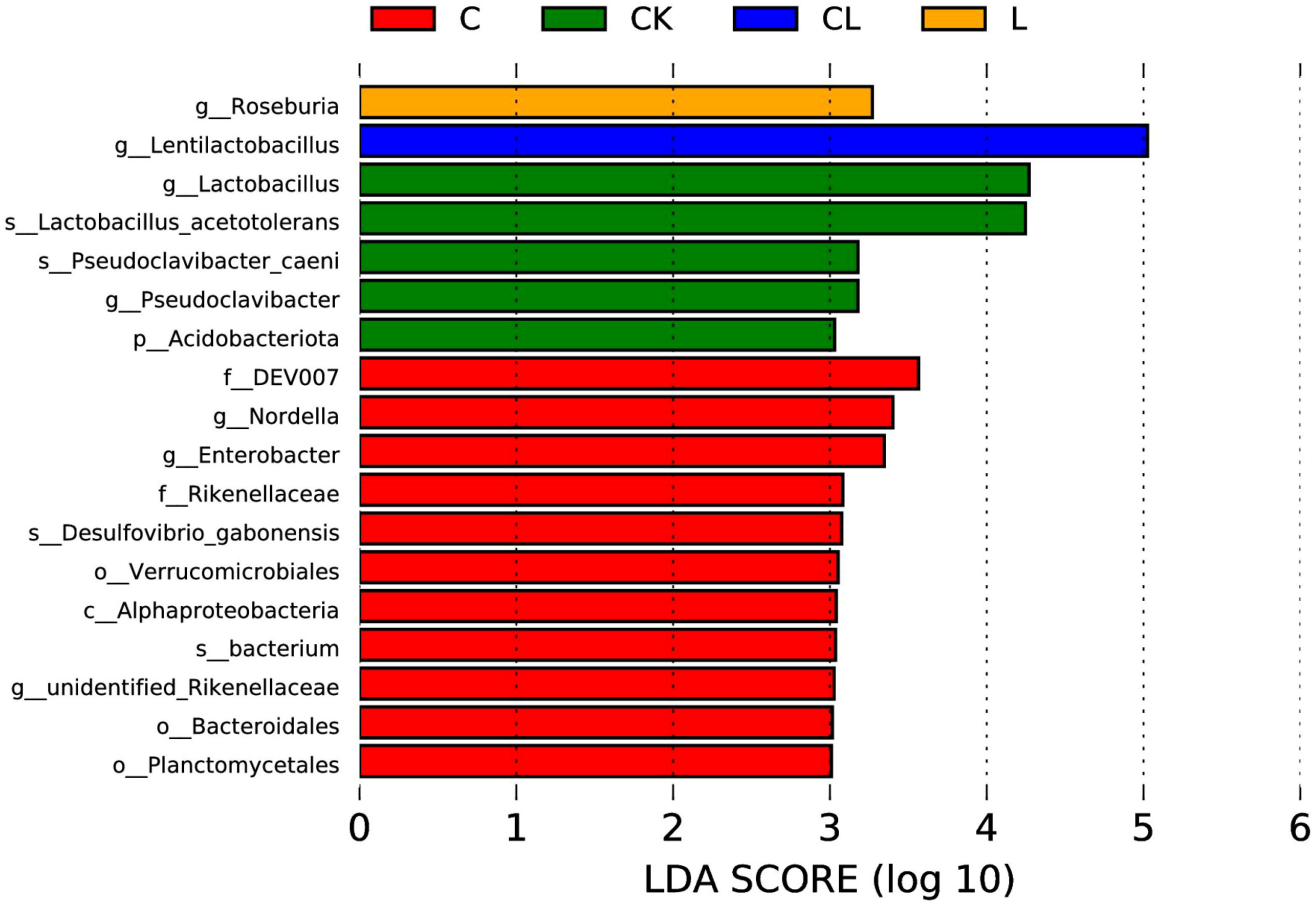
Linear discriminant analysis effect size (LEfSe) in each silage group (LDA score  >  3.0).

LEfSe analysis (LDA=3) revealed significant taxonomic differences between silage groups (**Figure 5**). After ensiling, g:_*Lactobacillus* was enriched in CK, g:*Nordella* in C, g:*Roseburia* in L, and g: *Lentilactobacillus* in CL.

## 4 Discussion

### 4.1 Chemical composition after ensiling

During process of silage fermentation, we observed no significant loss of DM in any of the experimental groups. Previous work identified WSC content as the main limiting factor for fermentation (Zhang et al., 2010). If the WSC content is low, then this will slow down the fermentation process of LAB, meaning that there will be no timely reduction of the silage pH, thus effectively inhibiting the activity of harmful microorganisms. In this study, we found that the WSC content of the treated groups was higher than that in the control group, and that the WSC content of the C and CL groups were significantly higher than that in the CK group, thus meaning better quality silage.

A reduction in fiber content fraction (NDF, ADF and ADL) can influence the cellulose content of silage; hemicellulose and lignin are decomposed during the silage process, thus releasing WSC which can provide more fermentation substrate for LAB and improve the fermentation quality of silage (Dean et al., 2005).

The CP content is an important indicator for assessing the nutritional value of feed; the higher the CP content, the higher the nutritional value of the feed (Lin et al., 2023). According to a recent report, adding 60 to120 U/g of cellulase to plant leaf silage can increase the contents of protein and other nutrients in silage (He et al., 2018). Consistently, a reporter observed an increase in CP contents of silages inoculated with the fibrolytic enzymes compared with their initial contents and attributed it to proteolysis inhibition (Moselhy et al., 2015). In our present study, CP in the CL group was significantly higher than in the other groups, and CP in C group was also significantly higher than in the CK and L groups. The lowest CP content in CK silage was consistent with the greatest AN content. These findings could be attributable to the cellulase mediated degradation of plant fibers, thus increasing the amount of soluble sugar available. This would provide more available substrates for silage fermentation and accelerate the propagation of lactic acid bacteria. This does not only inhibit the growth of harmful bacteria, but also inhibits the activity of plant protease in silage, thus reducing the hydrolysis of plant protein, and improving the utilization rate of nitrogen content in silage.

### 4.2 Fermentation quality after ensiling

The pH value is a key index used to evaluate the fermentation quality of silage and reflects the total acid content of silage; good quality has a pH of 3.8 to 4.2 (Weinberg, 2008). In our study, all additive-treated groups had a lower pH than the CK group, with all groups showing a pH < 4.2. The addition of C and L exerted a significant decrease on pH. Therefore, both cellulase and laccase accelerated the pH decline, and these two enzymes had synergistic effect.

The content of LA and volatile fatty acids are important indices with which to evaluate the quality of silage (Selmer-Olsen, 1994). Silage in good quality usually has a high LA content and almost no BA production (Filya, 2023). According to different fermentation products, LAB fermentation can be divided into homotype and heterotype forms of fermentation. The main product of homotype fermentation is LA, while heterotype fermentation products are mainly LA, AA, ethanol and carbon dioxide. LA can reduce the pH of silage, thus inhibiting the growth of other non-desirable forms of microorganism, such as yeast, mold and clostridium. AA is mainly produced by heterogeneous LAB, which exhibits strong antifungal ability. An increase in LA/AA value indicates that LAB can increase homozymosis and produce more LA. BA is the product of the degradation of protein, glucose and lactic acid by spoilage bacteria and butyric acid bacteria. It is generally believed that the content of butyric acid in high-quality silage should be less than 1% (Yuan et al., 2012). In our current study, we did not detect any evidence for the presence of BA, thus indicating that harmful microorganisms had been inhibited during ensiling. As reported previously, mixing corn straw and soybean residue with 100 U/g of cellulase significantly increased the content of LA and AA after 56 d of ensiling (Zhao C. et al., 2021). Consistent with these results, in the present study, we found that the contents of LA and AA in experimental groups were higher than those in the control group. The LA/AA values of C, L and CL were higher than those in the CK group. These data demonstrate that the addition of cellulase and laccase can achieve better effects in corn stover and wet brewer’s grains after ensiling. The NH_3_-N/TN value can reflect the microbial degradation of protein and amino acid in silage; the smaller the ratio, the smaller the loss of nitrogen and the better the quality of silage (Zhao G. et al., 2021). As reported previously (Menardo et al., 2015), we found that the values of NH3-N/TN in the experimental groups were lower than those in the control group. These findings might indicate that cellulase and laccase increase the amount of substrate available for LAB fermentation, thus accelerating the accumulation of lactic acid, reducing the pH, and inhibiting the hydrolysis of protein by harmful microorganisms (Heinritz et al., 2012). Therefore, the application of C, L and CL exert positive effects on protein preservation, thus improving the fermentation quality of silage made from corn stover and wet brewer’s grains.

Cellulase and laccase can hydrolyze the cell walls of plants and degrade some structural carbohydrates of silage raw materials into monosaccharides or disaccharides, thus providing sufficient substrate for LAB reproduction. In this study, the addition of C exerted a significant effect on LAB; all of the treated groups had higher LAB populations than the CK group. The LAB of the CL group was significantly higher than that of the CK and L groups, and the LAB of the C group was significantly higher than that of the CK group. These data indicate that the addition of C exerts positive effects on the LAB population.

Generally, yeast is not beneficial for the silage process as it can cause secondary fermentation in the silage. Mold is the main harmful microorganism that causes aerobic spoilage of silage. If silage is not sealed well or not compacted, and an anaerobic environment is not achieved, then mold will grow in large numbers, decompose cellulose and plant cell wall components, decompose sugar and LA, and change the composition of nutrients. *E. coli* is the major competitor of LAB and is responsible for reductions in the amino acid content of silages, resulting in the loss of nutrients from silage (Rauramaa et al., 1987). These spoilage microbes are usually inhibited at a pH < 4.5. In the present study, we did not detect yeast, mold or *E. coli* in any of our samples, thus indicating that all groups of corn stover and wet brewer’s grains silage sealing and fermentation have better effects.

### 4.3 Microbial community after ensiling

The silage process is a complex microbial symbiosis system that features the participation of multiple microorganisms (Zhou et al., 2016). Therefore, it is of great significance to study the community composition of microorganisms in silage. An OTU is the same flag set for a given taxa, such as strain, species and genus. Alpha diversity refers to the diversity within a specific environment or ecosystem, mainly reflecting the richness and evenness of species and sequencing depth (Caporaso et al., 2012). In a previous study (Wang et al., 2020), the addition of cellulase reduced the Chao1 index and Ace index of alfalfa silage samples, and reduced the diversity of the microbial community in feed. In the present study, the lower number of OTUs, along with the lower Shannon, Simpson, Chao1 and Ace indices in CL silage compared to the CK, L and C silage after 30 d of ensiling might have been due to the relatively low pH values in inoculated silages, thus limited the growth of microbes (Méndez-García et al., 2015).

The closer the distance between two points, the higher the similarity of microbial community structure between two samples in the PCA result. Our analysis revealed that samples from different additive groups can be distinguished in a manner with the CK group. This indicates that there are certain differences in the composition of microbial communities between the control group and the experimental groups, particularly the CL groups. We inferred that the addition of cellulase and laccase created synergies in their bacterial communities in mixed silage containing corn stover and wet brewer’s grains.

Firmicutes dominate in silage, most of which are gram-positive and produce spores that are resistant to dehydration and extreme environments; most of these can degrade cellulose, starch, protein, and other macromolecular compounds (Cai et al., 1999). Much research has revealed that the majority of the bacterial community involved in lactic acid fermentation in silage belongs to the phylum Firmicutes (Pang et al., 2011; Ogunade et al., 2018). Proteobacteria play a major role in polysaccharide utilization or BA fermentation, the digestion of organic matter, and carbon and nitrogen cycling during anaerobic fermentation (Yuan et al., 2020). According to a previous study (Mu et al., 2020), the main forms of bacteria present in mixed silage prepared from high-moisture amaranth and rice straw were Firmicutes and Proteobacteria. In the current study, Firmicutes and Proteobacteria were the most dominant phyla in mixed silage; the CL silage had the lowest abundance of Proteobacteria when compared to other groups, followed by the L group. The observed increase of the dominant phylum Firmicutes/Proteobacteria ratio from 5.84 (CK) to 5.04 (C), 9.44 (L), and 37.60 (CL), indicates a higher quality of silage in the L and CL groups. To identify differentially abundant taxa among each group, a more stringent LEfSe analysis was performed. Results revealed that all differentially abundant taxa identified by LEfSe analysis were categorized into Firmicutes and Proteobacteria.

LAB is the main microorganism responsible for the production of good silage, and can inhibit the reproduction of spoilage bacteria and other harmful bacteria, thus preserving the quality of silage (Zhou et al., 2016). In the present study, *Limosilactobacillus*, *Lactiplantibacillus*, and *Lentilactobacillus* were identified as the top three genera. These are the three most common genera of LAB. The sum of the three genera in the different silages was as follows (from high to low): CL > L > C > CK. Based on phylum and genus levels, our analysis clearly showed an improvement in fermentation quality in the experimental group, especially the simultaneous addition of cellulase and laccase, as these likely increased the microbial fermentation substrate available in the silage. These results also demonstrate a synergistic effect on the microbial community arising from the simultaneous addition of cellulase and laccase.

## 5 Conclusion

Collectively, our analyses indicated that the addition of cellulase significantly increased CP, WSC, LAB counts, while significantly decreased the NDF, ADF content. The addition of laccase significantly reduced the ADL content. The combined addition of cellulase and laccase significantly increased the CP, WSC, LAB counts, and significantly decreased the pH value, NDF, ADF and ADL content, thereby improving fermentation quality. In addition, the application of cellulase and laccase increased the abundance of Firmicutes and LAB genera, and decreased the level of microbial diversity. In summary, the combined addition of cellulase and laccase further improved fermentation quality, thus providing a new approach for the proper preservation and utilization of the agro-industrial by-products (corn stover and wet brewer’s grains).

## Data Availability

The data presented in the study are deposited in the NCBI Sequence Read Archive (SRA) repository, accession number PRJNA1114908.
